# 16185 Iowa Implementation for Sustainability Framework: Specification and validation

**DOI:** 10.1017/cts.2021.547

**Published:** 2021-03-30

**Authors:** Laura Cullen, Kirsten Hanrahan, Stephanie W. Edmonds, Heather Schacht Reisinger, Michele Wagner

**Affiliations:** 1 University of Iowa Health Care; 2 University of Iowa Carver College of Medicine; 3 University of Iowa Hospitals & Clinics

## Abstract

ABSTRACT IMPACT: Framework is designed to aid selection of implementation strategies to promote adoption and sustainability of EBP to improve health care quality, safety and value. OBJECTIVES/GOALS: An application-oriented implementation framework based on Diffusion of Innovation theory, identified 81 strategies for clinician-use within four implementation phases. The goal of this research was to further specify strategies based on emerging implementation science and establish external validity. METHODS/STUDY POPULATION: An iterative mixed-methods process guided framework revisions. First, individuals (n=1,578) requesting use of the framework over the last seven years were sent an electronic questionnaire. Evaluation captured usability, generalizability, accuracy of phases, and implementation phases for each of 81 strategies. Second, nurses who use the framework pile sorted strategies for multidimensional scaling and hierarchical analysis using Anthropac software. Third, a panel of five EBP/implementation experts used data and a consensus process to add clarity with the naming, and further specify strategies. RESULTS/ANTICIPATED RESULTS: Survey respondents (n = 127, 8% response) were nurses (94%), at least Master’s educated (94%), from health systems (52%) or academia (31%), in the U.S. (84%). The framework, rated on a four-point scale (1 = not/strongly disagree to 4 = very/strongly agree; reported are ratings 3 and 4) was deemed useful (92%), generalizable (100%), and with accurate timing (96%). 51 participants linked strategy timing to a single phase (54 strategies, 66.7%, p<0.05, Cochran’s Q); most strategies (30) matched the original model. Pile sorting (n=23) generated a concept map and hierarchical clusters of groups. Experts used these data and implementation science to specify each strategy and revise the framework. DISCUSSION/SIGNIFICANCE OF FINDINGS: The Iowa Implementation for Sustainability Framework (IISF) offers a typology to guide implementation for healthcare improvements. This study specifies 77 implementation strategies, confirms four phases, identified 10 domains, and begins to establish external validity for the framework.

